# Herpesvirus and Autophagy: “All Right, Everybody Be Cool, *This Is a Robbery*!”

**DOI:** 10.3390/v9120372

**Published:** 2017-12-04

**Authors:** Marion Lussignol, Audrey Esclatine

**Affiliations:** Institute for Integrative Biology of the Cell (I2BC), CEA, CNRS, University of Paris-Sud, Université Paris-Saclay, 91198 Gif-sur-Yvette CEDEX, France; marion.lussignol@u-psud.fr

**Keywords:** autophagy, subversion, herpesvirus, autophagosomes, amphisome, envelopment, egress, latency, reactivation, innate immunity

## Abstract

Autophagy is an essential vacuolar process of the cell, leading to lysosomal degradation and recycling of proteins and organelles, which is extremely important in maintaining homeostasis. Multiple roles have been now associated with autophagy, in particular a pro-survival role in nutrient starvation or in stressful environments, a role in life span extension, in development, or in innate and adaptive immunity. This cellular process can also take over microorganisms or viral proteins inside autophagosomes and degrade them directly in autolysosomes and is then called xenophagy and virophagy, respectively. Several Herpesviruses have developed strategies to escape this degradation, by expression of specific anti-autophagic proteins. However, we are increasingly discovering that Herpesviruses hijack autophagy, rather than just fight it. This beneficial effect is obvious since inhibition of autophagy will lead to decreased viral titers for human cytomegalovirus (HCMV), Epstein-Barr virus (EBV) or Varicella-Zoster virus (VZV), for example. Conversely, autophagy stimulation will improve viral multiplication. The autophagic machinery can be used in whole or in part, and can optimize viral propagation or persistence. Some viruses block maturation of autophagosomes to avoid the degradation step, then autophagosomal membranes are used to contribute to the envelopment and/or the egress of viral particles. On the other hand, VZV stimulates the whole process of autophagy to subvert it in order to use vesicles containing ATG (autophagy-related) proteins and resembling amphisomes for their transport in the cytoplasm. During latency, autophagy can also be activated by latent proteins encoded by different oncogenic Herpesviruses to promote cell survival and achieve long term viral persistence in vivo. Finally, reactivation of gammaherpesvirus Murid Herpesvirus 68 (MHV68) in mice appears to be positively modulated by autophagy, in order to control the level of inflammation. Therefore, Herpesviruses appear to behave more like thieves than fugitives.

## 1. A Brief Introduction to Herpesviruses

Herpesviruses were originally classified into a single family, but since 2009 they are grouped into the new order *Herpesvirales* and organized into three families [1]. They correspond to over two hundred different viruses identified to date, which infect a diverse array of animals: mammals, such as cattle, swine or horses, but also birds and reptiles (the *Herpesviridae* family) or fishes and amphibians (the *Alloherpesviridae*), and even mollusks (the *Malacoherpesviridae*) [2]. Among members of *Herpesviridae* family, nine have humans as their primary host and are present all around the world, with a high prevalence for most of these viruses. Herpesviruses have a common structure, a toroid-shaped DNA genome surrounded by an icosahedral capsid with 162 capsomers, a proteinaceous tegument and an outer lipid bilayer envelope containing glycoprotein spikes on its surface [2]. The herpesvirus cycle is characterized by two distinct phases: latency and productive (or lytic) cycle. Indeed, they do not survive outside the host for a very long time, but they have the ability to establish latency in different cell types and to persist in the host throughout its life. In cells harboring latent virus, no active viral production occurs, but the viral genome is maintained in the nucleus generally in the form of an episome, a closed circular molecule from which only a small subset of viral genes is expressed [3,4,5,6]. During latency, cells are modified by latent proteins but also by non-coding RNA, and improved cell survival is notably observed [7]. Regularly, the infectious productive cycle can resume and lead to virion production and, eventually, to the onset of a clinical disease. Reactivation is the process by which a latent virus switches to a lytic phase of replication [2].

The *Herpesviridae* family is divided into three subfamilies, based on biological properties. The Alphaherpesvirinae subfamily is defined on the basis of a variable host range, relatively short reproductive cycle resulting in rapid destruction of infected cells, and the establishment of latent infections primarily in sensory ganglia. This subfamily incorporates, for example, Herpes simplex virus type 1 and 2 (HSV-1 and HSV-2), Varicella-Zoster virus (VZV), and Duck enteritis virus (DEV). Members of Betaherpesvirinae subfamily have a restricted host range, a long reproductive cycle and are represented by Human Cytomegalovirus (HCMV) and Human Herpesvirus 6 (HHV6) in humans. The Gammaherpesvirinae subfamily contains the *Rhadinovirus* genus, which includes Kaposi’s sarcoma-associated herpesvirus (KSHV or HHV8), Rhesus monkey Rhadinovirus (RRV) and Murid Herpesvirus 68 (MHV68), and the *Lymphocryptovirus* genus, which includes Epstein-Barr virus (EBV) and related primate viruses. They are mainly hosted by primates and latency is ordinarily established in lymphoid tissues. They can replicate in vitro in lymphoblastoid cells and are associated with lymphoproliferative diseases.

**Figure 1:**
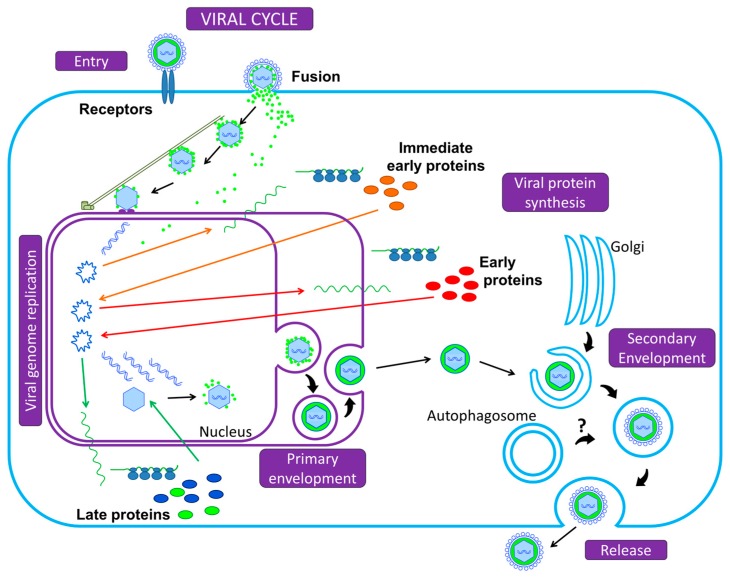
Summary of the Herpesvirus replication cycle. Initiation of infection begins with the adsorption of the viral particle to cell surface receptors and the virus enters the cell either by fusion of its envelope with the plasma membrane or by endocytosis, releasing the capsid and the tegument into the cytoplasm. Using the microtubule network, the nucleocapsid is transported to the nuclear pore, where the viral genome is released into the nucleus and circularizes. The viral DNA serves as a template for Pol II and leads to the production of mRNAs, expressed in three successive and coordinated phases. mRNAs are translated in the cytoplasm into viral proteins (α, or immediate-early; β, or early; and γ, or late proteins). Most of the late gene products contribute to the formation of the viral particle. DNA packaging into pre-assembled capsids takes place in the nucleus. This is followed by a primary envelopment of the nucleocapsids by budding through the inner nuclear membrane. The envelope of perinuclear virions then fuses with the outer nuclear membrane to release naked nucleocapsids into the cytoplasm (de-envelopment). Tegumented capsids acquire a “second” final envelope to become virions from post-Golgi membrane compartments. A role for autophagic membranes in envelopment and release of virions has been proposed for some herpesviruses. Once formed, virions are translocated to the cell surface within small vesicles using exocytosis machinery and released from the cells.

Finally, let us take a bird’s eye view of the Herpesvirus infectious cell cycle (Figure 1). To initiate infection, the viral particle attaches to cell surface receptors and rapidly enters the cell either by fusion of its envelope with the plasma membrane, or by endocytosis: either mode depends on the viruses and the cell types [2]. The tegumented capsid is then transported along the cytoskeleton into the cytoplasm to the nuclear pores, where DNA is released into the nucleus and is rapidly associated with histones. Transcription and replication of the viral genome take place in the nucleus, followed by assembly of nucleocapsids. Transcription into messenger RNAs (mRNAs) occurs using the cellular transcriptional machinery (in particular the host DNA-dependent RNA polymerase II) in an ordered cascade. Three classes of genes are sequentially and coordinately expressed (α, or immediate-early IE; β, or early E; and γ, or late L genes). IE gene products are required for the transcription of the E genes, which encode the DNA replication machinery. IE and E proteins, along with DNA replication, are required for efficient L transcription. Most of the L proteins are viral structural proteins or required for viral assembly. A primary envelopment of the nucleocapsid occurs at the inner nuclear membrane. This first envelope is then lost upon fusion with the outer nuclear membrane. Un-enveloped capsids are released into the cytoplasm and acquire tegument and final envelope by budding into specialized vesicles containing the viral glycoproteins that will decorate the surface of mature virions. The precise identity of the cytoplasmic compartment where this secondary envelopment occurs has not yet been resolved, but viral envelopes likely derive from membranes of the secretory or endocytic compartments (ER Golgi Intermediate Compartment/Trans Golgi Network/endosomal/exosomal membranes) [8,9]. The profound reorganization of the cytoskeleton and secretory/endocytic pathways during the infection blurs the lines [10,11,12,13]. The viral envelope membranes are likely to be heterogeneous and defining the precise origin of the cytoplasmic compartment is an unresolved question. Several studies recently reported a possible subversion of autophagic membranes for acquisition of the final envelope for at least two herpesviruses [14,15]. This will be detailed in Section 3.2 and Section 3.3 for EBV and VZV, respectively. Finally, the enveloped particle is transported in this specialized vesicle that later fuses with the plasma membrane, or spreads from cell to cell across cell junctions.

## 2. Autophagy

### 2.1. Background

Autophagy allows the cell to discard different cell components, from macromolecules to organelles. Although discovered more than 50 years ago—the word “autophagy” was invented by the Belgian biochemist Christian de Duve in 1963—we are still in the process of learning about the incredible resources of this essential cellular mechanism [16]. In order to function, autophagy requires a conserved and complex machinery, made of ATG (autophagy-related) proteins, initially discovered in yeast by pioneering researchers, such as Yoshinori Ohsumi, Michael Thumm, and Daniel Klionsky. Yoshinori Ohsumi was awarded the 2016 Nobel Prize in Physiology or Medicine for his discoveries on molecular and cellular mechanisms of autophagy [17]. Initially thought as a non-selective degradation process, autophagy turned out to be an extraordinarily sophisticated mechanism, highly selective for structures, organelles, or macromolecules. The three main types of autophagy in mammals are macroautophagy, microautophagy, and chaperone-mediated autophagy, depending on the mechanism that mediates the delivery of intracellular components to lysosomes. Only relationships between macroautophagy (hereafter referred as autophagy), the best-characterized autophagic process, and viruses have been uncovered so far. Macroautophagy degrades cytosolic components upon their sequestration in a double membrane vesicle, termed the autophagosome, which fuses with the lysosome to form the autolysosome. An endosome can also fuse with an autophagosome in certain circumstances to generate an amphisome, a single-membrane compartment, proving the tight relationship between endocytic and autophagic pathways [18]. Upon fusion of autophagosomes and amphisomes with lysosomes (to form autolysosomes), the content of the vacuole is quickly degraded by specific lysosomal enzymes and recycled. The autophagosome is formed by the closure of a structure called the phagophore, an expanding cup-shaped double-membranes structure [19]. Although the starting point of autophagosome formation seems to be an endoplasmic reticulum (ER) subdomain called “omegasome”, mitochondria, lipid droplets, ERGIC vesicles, Golgi, endosomes, and ER-plasma membrane contact sites also participate in autophagosome biogenesis [19,20]. So far, over 30 ATG genes have been identified in yeast, and eighteen ATG proteins are engaged in autophagosome formation [21]. Many of these genes have homologs or functional analogs in multicellular organisms [22]. In mammals, the ATG8 family LC3 (microtubule-associated protein 1 light chain 3)/GABARAP is central in the autophagic pathway, acting both in autophagosome formation and maturation [22]. The lipidated form of LC3 (LC3-II) is widely used as marker for autophagosomes and generally accumulates when autophagy is activated.

Autophagosomes can engulf cytoplasm indiscriminately and contain non-specific cargoes. This random process is the so-called non-selective “bulk” autophagy, whereas selective autophagy corresponds to specific engulfment of intracellular portions and cargoes, which are recognized by autophagy receptors. Among these receptors, p62 or sequestosome1/SQSTM1 interacts with cargoes, such as ubiquitinated damaged mitochondria or intracellular bacteria, to target them to autophagosomes. Via their interaction with autophagy receptors, LC3s/GABARAPs also function during selective autophagy. Autophagy is a highly dynamic, multi-step process. Like other cellular pathways, it can be modulated at several steps, both positively and negatively. We have to be aware that accumulation of LC3-II positive autophagosomes does not always reflect an increased autophagic activity, but also a decreased clearance consecutive to a block of the autolysosomal degradation. Since SQSTM1 and LC3 are degraded by autophagy, their turn-over can be used as indicators of the autophagic flux (autophagic degradation activity).

### 2.2. Physiological Cellular Roles, Defense Mechanism, Innate and Adaptive Immunity

Autophagy acts in numerous processes, such as cellular homeostasis, quality control and cell survival. This cellular process ensures quality control of proteins and organelles, by clearing toxic components generated by the cells, such as damaged mitochondria (thus limiting the production of reactive oxygen species) or accumulation of aggregation-prone proteins. Autophagy thus plays an important role against neurodegenerative diseases, by clearing toxic abnormal protein aggregates. The autophagic pathway can also be stimulated in response to various forms of cellular stress, from nutrient deprivation to hypoxia, from chemical and physical stress to endoplasmic reticulum (ER) stress, and constitutes a major pro-survival cell program [23]. Even in the absence of stress signals, normal cells require autophagy to maintain homeostasis. Additionally, autophagic activity decreases with age in many tissues and organs [24]. Recent evidence indicates that sustained autophagy by nutritional, pharmacological or genetic approaches can extend the lifespan and/or health span of animals, from flies to mice [25]. Autophagy contributes to development and specifically eliminates, for example, paternal mitochondria in the embryo after fertilization [26].

The functional relevance of autophagy regarding tumorigenesis and tumor progression is more complex, although extensively studied, and is likely dependent on tumor stage [27]. Indeed, autophagy can exert important tumor-suppressive functions, frequently observed at the initial stage of cancer development. Several tumor suppressor genes and oncogenes affect autophagic pathways and there is a direct relationship between decreased autophagic activity and tumorigenesis. Autophagy prevents transformation, by recycling damaged proteins and organelles, by limiting necrosis and inflammation and facilitating oncogene-induced senescence. In contrast, once a tumor is formed, autophagy may promote tumor progression and metastasis because it functions as a survival mechanism which provides adaptive response to stress [28]. Induction of autophagy facilitates survival of tumor cells which are under nutrient-limited and low-oxygen conditions, by maintaining energy homeostasis in their microenvironment. Autophagy also provides resistance to anoikis (detachment-induced cell death) and apoptosis induced by anticancer treatment. Autophagy inhibitors can restore the sensitivity to chemotherapy-mediated tumor cell death, therefore, regulators may be exploited in the future as a potential therapeutic approach against cancer.

The roles of autophagic pathways in innate and adaptive immunity are essential [29]. Autophagy controls inflammation by limiting inflammasome activation and by degrading inflammasome components, such as NLRP3 (NOD-like receptor family, pyrin domain containing 3) and AIM2 (absent in melanoma 2). Autophagy also inhibits type I interferons production, which are powerful antiviral cytokines, and it regulates NF-κB (Nuclear factor-kappa B) activation, involved in the production of several pro-inflammatory mediators. Thanks to the autolysosome vesicles, which allow degradation of large cargoes, autophagy can also directly eliminate intracellular microbes and can engulf apoptotic cells [30,31]. Some bacterial pathogens, including group A *Streptococcus*, *Mycobacterium tuberculosis*, *Salmonella*, or *Listeria monocytogenes* have increased intracellular survival when autophagy is suppressed. This selective autophagy is called xenophagy. Few examples of real degradation of viral particles into autolysosomes have been reported. However, autophagic receptors, such as SQSTM1, can selectively target viral proteins to be degraded into the autolysosomes. The non-structural Tat protein of human immunodeficiency virus type 1 (HIV-1) is recognized by SQSTM1 in CD4+ T lymphocytes and targeted for autophagic clearance, contributing to the restriction of HIV-1 [32]. Selective autophagy-mediated degradation of the capsid proteins of two Togaviruses, Sindbis virus and Chikungunya virus, relies on a ubiquitin-dependent interaction with SQSTM1 [33,34]. Deletion of autophagy results in a failure to clear Sindbis viral capsid protein and increases cell death [33]. Peptides generated from proteins degraded by autophagy can also be used for antigen presentation to T-cells by both Major Histocompatibility Complex (MHC) class I and II, for regulation of immunity and host defense [35]. This catabolic pathway can deliver via autophagosomes endogenous viral antigens, such as the EBV-encoded Epstein-Barr Nuclear Antigen 1 (EBNA1) latent protein, for MHC class II presentation and it can also facilitate the processing and the loading of the HSV-1 gB glycoprotein onto MHC I molecules [36,37,38].

### 2.3. Herpes Viral Escape

Given that autophagy is involved in innate and adaptive immunity and described as an antiviral mechanism, it is not surprising that viruses encode proteins to counteract this process, especially in Herpesviruses, which are highly adapted to their hosts [39]. Several anti-autophagic proteins have already been described, for HSV-1, HCMV, Herpesvirus Saimiri, and HHV8 [39]. Historically, the first anti-autophagic viral protein to be discovered was a neurovirulence gene product, ICP34.5 encoded by HSV-1 [40]. Control of autophagy by ICP34.5 is mediated by its capacity to bind Beclin1/BECN1. BECN1, the mammalian homolog of ATG6, is an essential component of the autophagic machinery, present with class III phosphatidylinositol 3-kinase in two complexes involved in the biogenesis and the maturation of autophagosomes. While a virus lacking *ICP34.5* genes triggers autophagy by activating the EIF2AK2/PKR (eukaryotic translation initiation factor 2-α kinase 2) pathway, ICP34.5 is able to control autophagy by interaction with BECN1, and this is mandatory for HSV-1 neurovirulence [41]. Autophagy activation protects adult brains from viral encephalitis, but this protection is age-dependent, since it seems that in newborn mouse brains it is deleterious and promotes apoptosis [42]. Another way for the cell to detect HSV-1 and to trigger autophagy occurs via recognition of cytosolic HSV-1 DNA by the cGAS DNA sensor and BECN1 and partially by STING (Stimulator of Interferon Genes), leading to the delivery of DNA to autophagosomes [43,44]. It is interesting to note that autophagy does not seem to have the same impact on HSV-1 infection in all cell types. Indeed, autophagy is critical for viral control in primary neurons in vitro, while it is dispensable in fibroblasts (the virus replication is not modified in ATG5-deficient fibroblasts) [45,46]. In mice, this difference between cell types is also observed: autophagy is involved in antiviral defense in neurons, but not in epithelial cells [46]. In fibroblasts, in neurons, and in epithelial cells, ICP34.5 inhibits the autophagosome biogenesis. However, the effect of ICP34.5 on autophagy is different in professional antigen presenting cells. Indeed, in dendritic cells (DCs), ICP34.5 blocks the maturation of autophagosomes. This inhibition has a role in immune escape, by reducing viral antigen presentation by DCs [47].

It has been described that each herpesvirus genomes can encode several anti-autophagic proteins, acting on different stages of autophagy. In addition to ICP34.5, the Us11 protein, expressed later in HSV-1 infection than ICP34.5, can inhibit autophagy by directly interacting with PKR [48]. HCMV encodes two highly homologous proteins, called IRS1 and TRS1, which bind to BECN1 to inhibit autophagy. Interestingly, whereas synchronous expression of these two proteins blocks the maturation of autophagosomes, they both inhibit the initiation of autophagy when expressed separately [49,50]. HHV8 encodes no fewer than three anti-autophagic proteins, vFLIP (viral FADD-like interleukin-1-β–converting enzyme-inhibitory protein) vBcl-2 and K7, targeting different actors in the autophagic machinery [51,52,53]. Inhibition of autophagy by HHV8 could participate to the immune suppression induced by the virus [54]. Indeed, HHV8 infection of monocytes inhibits their differentiation into DCs by blocking autophagy, which has been described as necessary for monocyte differentiation [55,56].

Autophagy is also involved in the cellular response to restrict propagation of animal Herpesviruses. Pseudorabies virus (PRV), a swine alphaherpesvirus, is of great interest for virologists, as it serves as a model organism to study herpesviruses and infects a large range of vertebrates [57]. It is the etiological agent of Aujeszky’s disease, associated with respiratory and central nervous system afflictions. It has been described that inhibition of autophagy seems to improve viral titers of PRV [58]. PRV modulates autophagy and the authors have identified a viral protein candidate called Us3 that might be involved in PRV counterattack against autophagy. Autophagy is also well-conserved in oysters [59]. They express ATG proteins which are closer to human proteins than those of flies or nematodes. Oysters can be infected by Ostreid Herpesvirus 1 (OsHV-1), a virus responsible for recent mass mortality outbreaks in oyster cultures, and autophagy plays a protective role against this infection [59,60]. Indeed, autophagy stimulation is associated with an increase in oyster survival to OsHV-1 infection.

To conclude, numerous studies describe different strategies developed by Herpesviruses to escape the degradative process, but, to date, it is not completely established whether this cellular mechanism plays a major role in fighting viral infection. Visualization by electronic microscopy of Δ34.5 mutant HSV-1 virions inside autophagosomes suggests that HSV-1 particles could be degraded by virophagy [61]. Autophagy was also initially proposed as a way for the cell to get rid of HCMV [62]. However, although the anti-autophagic HCMV proteins TRS1 and IRS1 are essential for the virus to propagate, it is not related to their ability to block autophagy [63]. Similarly, HHV8 vBcl-2 is required for viral replication, not because of its anti-autophagic and anti-apoptotic properties but, in fact, because of an uncharacterized nuclear function [64,65].

## 3. Autophagy Subversion by Herpesviruses

Several years ago, the idea emerged that autophagy is not just a defense mechanism against pathogens, but can also be exploited by viruses to enhance their multiplication or improve their persistence during latency. For some Herpesviruses, the maturation step of autophagy is blocked, impeding the lysosomal degradation and allowing the exploitation of autophagic membranes for assembly and the release of the viral particles. Other viruses take advantage of the degradation functions of autophagy, for example, in order to selectively degrade antiviral molecules. Autophagy can also participate in the persistence of Herpesviruses during latent infection by improving cell survival. Moreover, activation of autophagy in EBV-infected cell lines, by delaying cell death, plays a role in the immortalized and transformed phenotype of these cells [66].

### 3.1. Autophagy Promotes Viral Production of Several Herpesviruses

As described above, regulation of autophagy by HSV-1 is cell type dependent, but most of the studies observed a detrimental role of autophagy during HSV-1 infection, either by improving antigen presentation, or by decreasing viral multiplication. A recent paper reported a transient induction of autophagy by HSV-1 in human monocytic THP-1 cells, which appeared to have a proviral role [67]. Indeed, pretreatment of cells with autophagy inhibitors and infection at a high multiplicity of infection (MOI) leads to a decrease in viral titers. Similarly, HSV-1 replicates less in BECN1-knockdown THP-1 cells. Autophagy may be beneficial for viral entry, by a mechanism that still needs to be uncovered [67]. The activation of the autophagic pathway occurs rapidly and coincides with the interaction between virions and the cell surface, possibly via Toll-like Receptors (TLRs). Binding of HSV-1 to the cell surface, via TLR2 and TLR9, triggers the recruitment of the myeloid differentiation primary response (MyD88) adaptor protein, leading to NF-κB activation [68]. Induction of autophagy by HSV-1 in THP-1 cells involved MyD88, since infection of MyD88-deficient cells does not trigger autophagy. Infection of rabbit corneal cells by HSV-1, but also by HSV-2 leads also to an activation of autophagy, which could be protective against apoptosis [69]. HSV-1 and HSV-2 are closely related viruses sharing around 50% of sequence identity. They both encode ICP34.5 and Us11, but the role of this two proteins regarding autophagy during HSV-2 infection has not been studied yet. As observed with HSV-1, autophagy seems to be controlled in HSV-2-infected fibroblasts but no viral protein able to repress autophagy has been identified yet [70]. Treatment with bafilomycin, an inhibitor of the autophagic flux, decreased HSV-2 replication, suggesting that the virus benefits from functional autophagy to propagate in fibroblasts. These results were confirmed in ATG5-deficient cell lines, in which the virus replicated less efficiently. It is interesting to note that similar experiments performed in ATG5-deficient murine fibroblasts using HSV-1 did not lead to the same results, since no significant impact on the virus replication was observed [45]. How HSV-2 uses autophagy needs to be explored.

VZV and DEV also belong to the Alphaherpesvirinae subfamily, and both viruses activate autophagy, at late times during infection, and use this mechanism to optimize their propagation [71,72]. DEV causes severe illness in ducks, swans, and geese worldwide, characterized by vascular damage, hemorrhage, organ necrosis, and sudden death, and therefore represent an economic burden [73]. A recent publication focused on studying the impact of DEV on autophagy, and vice versa [72]. Infection of duck embryo fibroblasts (DEF) by DEV leads to an increase of autophagic flux after 24 h of infection. This induction of autophagy relies on viral protein expression, because it happens late in the viral life cycle and is not triggered by heat-inactivated virus. Inhibition of autophagy using drugs or siRNA against BECN1 and ATG5 decreased viral titers, and conversely treatment with rapamycin increased viral production. The differences in terms of viral titers were slight, but statistically significant, which means that even though autophagy seems to participate in the viral life cycle, it is not mandatory. The proviral role of autophagy during VZV infection has been the subject of several publications and will be developed further below in Section 3.3.

Autophagy is not only beneficial for several alphaherpesviruses, but the betaherpesvirus HCMV also subverts it for its own profit. We and others have reported that HCMV stimulates autophagy early after infection of fibroblasts and that components of the viral particles, such as viral DNA, are sufficient to trigger this mechanism [50,74]. However, later on, the autophagic flux is blocked by the virus, thanks to TRS1 and IRS1 [49]. A mutant virus lacking the ability to block autophagy still replicated similarly to the wild-type (WT) virus. More interestingly, the use of different autophagy inducers enhanced HCMV production, while treatment with SPAUTIN (Specific and Potent Autophagy Inhibitor) decreased viral titers. Therefore, it is likely that HCMV uses autophagic proteins or membranes for its propagation. However, these results are in contradiction with a study published in 2015 reporting that trehalose, a natural disaccharide initially reported to be pro-autophagic, inhibits HCMV replication, possibly by triggering autophagy [75]. While the antiviral activity of trehalose on HCMV is robust and can be reproduced in our hands, the link with autophagy still needs to be demonstrated. Moreover, long term treatment with trehalose might lead to unspecific effect on the cell metabolism and recent evidence demonstrates that trehalose blocks autophagic flux after 24 h of treatment [76]. Understanding which steps of the herpesvirus cycle are facilitated by autophagy should be a major research focus for Herpesvirologists.

### 3.2. During Lytic Cycle, EBV, and HHV8 Inhibit the Autophagic Flux and Exploit the Autophagic Vacuoles for Their Transport or Their Egress

It has been reported in at least three different studies that EBV lytic cycle induction in B cells is associated with an increased number of LC3-positive vesicles in the cytoplasm [14,77,78]. This accumulation of autophagosomes results from incomplete autophagy, by inhibition of the fusion between autophagosomes and autolysosomes [14,78]. Whereas the inhibition of the maturation step is clearly related to the expression of one or several lytic viral proteins, shadows zones persist to clearly identify the viral actors. Hung and collaborators observed that ectopic expression of BRLF1/Rta, a viral transactivator normally expressed during the immediate-early stage of the lytic cycle, induces a complete autophagic flux by a transcriptional mechanism, whereas Zebra (another immediate-early protein also denoted BZLF1 or Zta) does not, per se, modulate autophagy [77]. However, Granato and collaborators, the same year, observed a different phenotype regarding BZLF1/Zebra [78]. Transfection of a plasmid encoding BZLF1 stimulates autophagy in EBV-negative epithelial cells but is associated with an inhibition of the autophagic flux in the same cell line transfected with complete EBV genome. Interestingly, transfection of BZLF1 in EBV-positive cells induces the lytic cycle and expression of the complete set of EBV lytic genes. Therefore, the viral protein(s) involved in the inhibition of the autophagic flux are yet to be identified. A decreased expression of Rab7, a member of the family of GTPase proteins (guanosine-5′-triphosphatase) which has been implicated in the late maturation of autophagosomes [18], could contribute to the autophagic block in the context of EBV reactivation [78].

**Figure 2:**
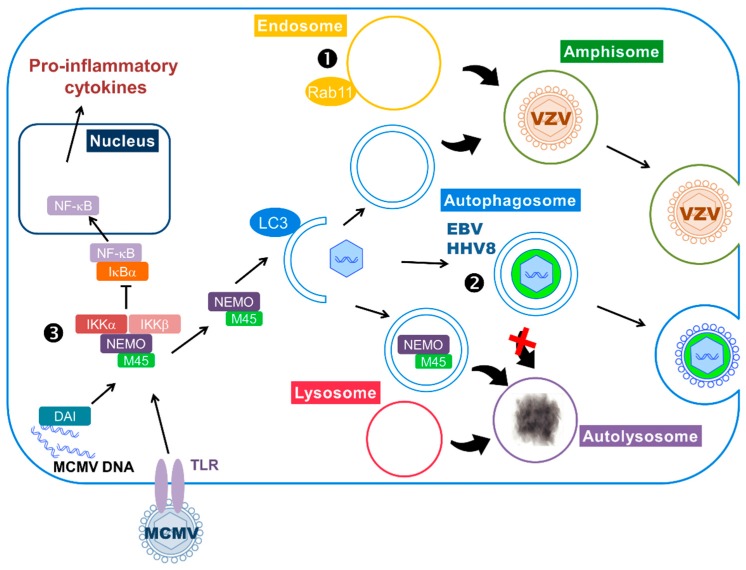
Subversion of autophagy during the lytic cycle. ➊,➋ Herpesviruses can use autophagy-derived vesicles for their transport within the cytoplasm and their release. ➊ VZV particles are incorporated in amphisome, a vacuole resulting from the fusion of autophagosome and endosome. ➋ Autophagosomes transport HHV8 and EBV particles to the cell surface. The viruses block autophagosome fusion with the lysosome to avoid degradation (red×). Moreover, EBV uses autophagosomal membrane for secondary envelopment and LC3 can be found in the virion. ➌ MCMV infection triggers the inflammatory cascade, via activation of TLRs and DAI (DNA-dependent activator of interferon regulatory factors), leading to nuclear translocation of NF-κB and transcription of pro-inflammatory cytokines. The viral protein M45 targets NEMO (NF-κB Essential Modulator), a protein involved in the activation of NF-κB, and redirects it to autophagosomes, where it will be degraded after fusion with the lysosome.

Using this strategy, EBV may limit lysosomal degradation of viral components and hijack the autophagic vesicles for its own profit (Figure 2). Indeed, genetic or pharmacological inhibition of autophagy reduces EBV lytic gene expression and viral production [14,78]. Autophagy modulation has no effect on replication of the viral genome but leads to its sequestration in the cytosol [14]. Co-purification and morphological analysis revealed the presence of lipidated LC3-II in viral particles released in the supernatant, suggesting that EBV may incorporate autophagic membranes into viral particles during its secondary envelopment in the cytosol. Alternately, it has been proposed that the autophagic vesicles could be rerouted to the plasma membrane in order to transport viral particles [78]. Contrary to these findings, a recent study reported that the knockdown of BECN1 increases viral lytic gene expression and EBV replication, suggesting that autophagy hampers EBV reactivation [79]. The authors also reported that, unlike the previous studies, EBV early lytic proteins blocks autophagosome formation. Therefore, the latter study will need to be confirmed.

Another gammaherpesvirus has been reported to accumulate autophagic membranes during lytic cycle in order to facilitate the transport of viral particles into the cytosol [80]. Indeed, it has been shown that reactivation of HHV8 from primary effusion lymphoma cells activates autophagy, leading to an increase in the number of autophagic vacuoles [80,81]. Similarly to EBV, HHV8 could block autophagic flux by downregulation of Rab7. Here again, knockdown of autophagy reduces HHV8 reactivation from latency. Ultrastructural studies suggest that autophagy is hijacked to allow the transport of viral particles into autophagosomes [80].

Such inhibition of maturation leading to an accumulation of autophagic membranes for the benefit of the viruses is not limited to Herpesviruses. Indeed, similar hijacking was also shown for influenza A virus (IAV) and Parainfluenza virus 3 (PIV3), two enveloped RNA viruses [82,83]. IAV could use LC3-enriched plasma membranes for filamentous budding of virus particles and for stability of viral progeny [84]. Upon PIV3 infection, inhibition of the autophagic maturation step by the phosphoprotein P (which interacts with SNAP29) is crucial for optimal viral budding at the plasma membrane and release [85].

### 3.3. Stimulation of Functional Autophagy and Lytic Cycle

VZV induces complete autophagic flux to help viral propagation. Several studies have shown that VZV infection triggers autophagy, and that no counterattack is carried out by the virus against this cellular response [71,86,87]. One hypothesis explaining why VZV does not block autophagy, unlike HSV-1, is that it does not encode for IC34.5 or Us11 homologs, the two described anti-autophagy HSV-1 proteins. Both WT and attenuated strains of VZV stimulate autophagy in different cell types, at late time of infection. This stimulation was assessed by an increase conversion of LC3-I to LC3-II and decreased levels of p62/SQSTM1. Several observations made in vivo showed that autophagy is also induced in skin lesions caused by VZV. A very high level of autophagosomes is observed in skin biopsies from patients with varicella or zoster. In an animal model using severe combined immunodeficiency (SCID) mice, human skin xenografts inoculated with VZV showed accumulation of LC3 positive puncta [87]. Further analysis of infected fibroblasts by pulse chase experiments and tandem-tagged LC3 confirmed that the virus induced a functional autophagic flux. The mechanism of autophagy induction during VZV infection is not clearly understood, but one hypothesis is that it might be triggered secondary to the ER stress caused by the infection in order to maintain cellular homeostasis. There is evidence of ER stress activation during VZV infection, like an expansion of the ER, and Unfolded Protein Response (UPR), a combination of pathways that will help relieving the ER stress [88]. VZV glycoproteins ectopic expression is sufficient to induce both autophagy and ER stress.

In order to demonstrate a potential pro-viral role of autophagy in VZV infection, Buckingham and collaborators used different strategies to modulate autophagy and analyzed different viral parameters like infectivity and viral protein expression [89]. First, they used a pharmacological treatment with 3-methyladenine (3-MA) to inhibit autophagy. The choice of 3-MA might be discussed, since it is known to have a pro-autophagy effect when the treatment is too long [90]. Nevertheless, they observed less propagation of the virus and a decreased infectivity of the virions after 3-MA treatment and in MeWo cells expressing siRNA against ATG5. By performing analysis of infectivity fractions, they observed a decreased expression of gE, one of the VZV glycoproteins, after 3-MA treatment, and an increase after trehalose treatment. More interestingly, in ATG5-deficient cells, there is a difference in the molecular mass of gE and gI compared to WT cells. It appears that the processing of viral glycoproteins in autophagy deficient cells is less efficient, leading also to an accumulation of gE dimers. Since viral glycoprotein biosynthesis induces ER stress, one hypothesis is that autophagy could relieve the ER stress, allowing a correct glycoprotein processing.

Further studies from the same group showed that gE colocalizes with LC3 and with rab11, a marker of recycling endosomes [15]. They found the two proteins associated with VZV purified particles, by immunoblot and immune-electron microscopy. The viral-containing vesicles did not show features of autophagosomes, as they only have single membranes. These vesicles are very likely to be amphisomes, containing one or several viral particles. The authors proposed that theses amphisomes could be used as exocytic vesicles by the virus to be released from the cell (Figure 2).

The degradative function of autophagy is hijacked by MCMV to counteract inflammatory response. Interplay between autophagy and innate anti-viral immune response is complex: as explained in Section 2.2, autophagy can participate in the activation of IFN responses by helping the delivery of viral PAMPs (Pathogen-Associated Motif Patterns) to endocytic TLRs, and can also degrade viral components. On the contrary, in the case of murine cytomegalovirus (MCMV), the virus hijacks the degradative function of autophagy to counteract innate immunity [91] (Figure 2). MCMV is a betaherpesvirus commonly used as a model for HCMV infection, especially because it allows in vivo studies. Both MCMV and HCMV are able to regulate the inflammatory response triggered by their infection. One of their common targets is the transcription factor NF-κB, which can be activated by pro-inflammatory cytokines and PAMPs, like viral DNA. Activation of NF-κB leads to its translocation to the nucleus and transcription of pro-inflammatory cytokines. The activity of NF-κB is regulated by inhibitory proteins known as IκB proteins, under the control of IκB kinase complex (IKK) composed of NF-κB Essential Modulator (NEMO), IKKα and IKKβ. Activation of the IKK complex leads to the degradation of IκB proteins and, therefore, releases NF-κB that can translocate to the nucleus. After a transient activation of NF-κB in MCMV-infected cells, the virus blocks it by targeting different proteins of the pathway [92]. A viral protein encoded by the M45 gene has been identified as an important factor in this inhibition, as it is able to block NF-κB activation induced by different stimuli. M45 protein, also called vIRA (viral inhibitor of RIP activation), specifically interacts with NEMO and its expression induces the degradation of this IKK subunit [91]. The authors showed that the M45-dependent degradation of NEMO is performed by autophagy, since they observed a delocalization of NEMO in autophagosomes. Moreover, its subsequent degradation required a functional autophagy as demonstrated by a stable amount of the protein after infection of ATG5-deficient cells.

Interestingly, a study of autophagy during MCMV infection of retinal pigment epithelial cells (RPE) showed that, similarly to HCMV, MCMV induced autophagy at early time of infection and subsequently blocked it [93]. More specifically, in this cell type, MCMV appears to block the autophagic flux, leading to an accumulation of autophagosomes. This might be a way for the virus to escape virophagy, as the authors described viral particles inside autophagosomes. However, to our knowledge, no evidence demonstrates a deleterious effect of autophagy against MCMV in this cell type. In comparison, in murine fibroblasts, an accumulation of LC3 is quickly observed and progressively builds up [91]. This accumulation does not result of an inhibition of autophagosome degradation, since it is further increased by addition of a lysosomotropic agent. This is consistent with the degradation of NEMO by autophagy, which happens early after infection. How M45 is able to target NEMO to autophagosomes for selective degradation needs to be further studied.

### 3.4. Autophagy Can Be Enhanced during Latency to Favor Cell Survival and It Can Control Excessive Inflammation during Reactivation

Persistence in the host organism via latency is a defining property of all the Herpesviruses. Regulating autophagy can give the virus a way of maintaining latency, as autophagy is involved in the regulation of cell death. As a matter of fact, latency is frequently associated with anti-apoptotic activity and numerous interactions exist between apoptosis and autophagy. Each Herpesvirus establishes latency in a specific set of cells, with the cellular site of latency differing from one virus to another. No viral particle is produced during latency but a set of specific viral genes are expressed depending on the herpesvirus. HSV-1 probably expresses the simplest program of latency with virtually no viral proteins expressed and only latency associated transcripts (LAT) non-coding RNA whereas EBV is characterized by several latency programs contributing to cell proliferation. Until now, the involvement of autophagy in latency regulation has been reported only for gammaherpesviruses, namely EBV, RRV, HHV8, and MHV68. These four viruses establish latency in lymphoid cells and are oncogenic. It would be of great interest to determine whether alphaherpesviruses, such as HSV-1, or betaherpesviruses use autophagy for establishing latency or during reactivation. Perhaps this association between autophagy, latency, and tumorigenesis is not a coincidence, since only few Herpesviruses are oncogenic, in particular EBV and HHV8 for humans.

Enhanced autophagy in EBV-positive cells is associated with an improved cell survival and is dependent of latent membrane proteins expression. Latent EBV genomes express six EBV-encoded nuclear antigens (EBNA), three latent membrane proteins (LMPs), and diverse non-coding RNAs such as EBV-encoded small RNA (EBER), BART (BamHI-A region rightward transcript) and miRNAs [7]. The Bcl-2 homolog BHRF1 (BamHI fragment H rightward open reading frame 1), which is classically described as a lytic protein, is also expressed in some latency programs [94]. EBV latency gene expression varies in the different latency phenotypes, from latency 0 with no viral protein expression and latency I where only EBNA1 is expressed to latency III, where the 6 EBNAs and the 3 LMPs are expressed. Latent EBV infection can lead to lymphoproliferations and to B cell malignancies, including Burkitt lymphoma (BL) largely associated with latency I program and post-transplant lymphoma disorder (PTLD), most of the time associated with latency III.

It has been recently described that autophagy can be constitutively activated in EBV latent infection of B cells, depending on the latency programs [66]. Indeed, EBV-positive latency II and latency III B cells are associated with an enhanced level of basal autophagy, compared to EBV negative BL cell lines. This enhanced autophagy is associated with a higher resistance to cell death. Nutlin-3, a p53 inducer, can increase apoptosis and inhibit tumor growth in clinical trials. EBV positive latency III cells are however resistant to Nutlin-induced cell death. In these cells, level of autophagy is further enhanced by nutlin-3 treatment and this confers high resistance to cell death, whereas EBV negative cells similarly treated by nutlin-3 die massively through an apoptotic process. Autophagy is involved in the cell survival, because inhibition of autophagy by chloroquine treatment is able to restore Nutlin-3-induced apoptosis in EBV positive cells. The molecular mechanism of this stimulation is not completely identified, but BECN1 expression is upregulated and mTOR pathway is inhibited consequently to p53 activation.

Interestingly, autophagy is not enhanced in EBV positive latency I lymphocytes [66]. Whereas EBNA-1 is the only EBV latent protein associated with latency I, the full range of latent proteins is expressed in latency III B cells. Among the viral products, mostly LMP1 has been involved in tumor formation with an overall effect on cell survival. It was initially reported in 2008 that expression of LMP1 in B cells derived from an EBV negative BL can enhance the level of basal autophagy, depending of its level of expression [95]. Whereas cells that express low levels of LMP1 preferentially accumulate early autophagosomes, high levels of LMP1 expression induce an accumulation of autolysosomes, corresponding to later stages of autophagy. Furthermore, in cells expressing high levels of LMP1, degradation of LMP1 depends on autophagy, since the authors observed an accumulation of LMP1 in autophagy-deficient cells and this degradation seemed to be necessary for the proliferation of B cells. The mechanism of autophagy activation by LMP1 is controversial. One possibility is that it could be related to the property of LMP1 to phosphorylate the eIF2α kinase PERK (Protein kinase RNA-like endoplasmic reticulum kinase). However, it remains to be confirmed whether LMP1 is able to stimulate autophagy by PERK activation. Activation of autophagy by LMP1 was recently confirmed by Pujals and collaborators in EBV-positive B cells. Furthermore, the authors observed that LMP1 activation is NF-κB dependent, contrasting with the initial study [66].

**Figure 3:**
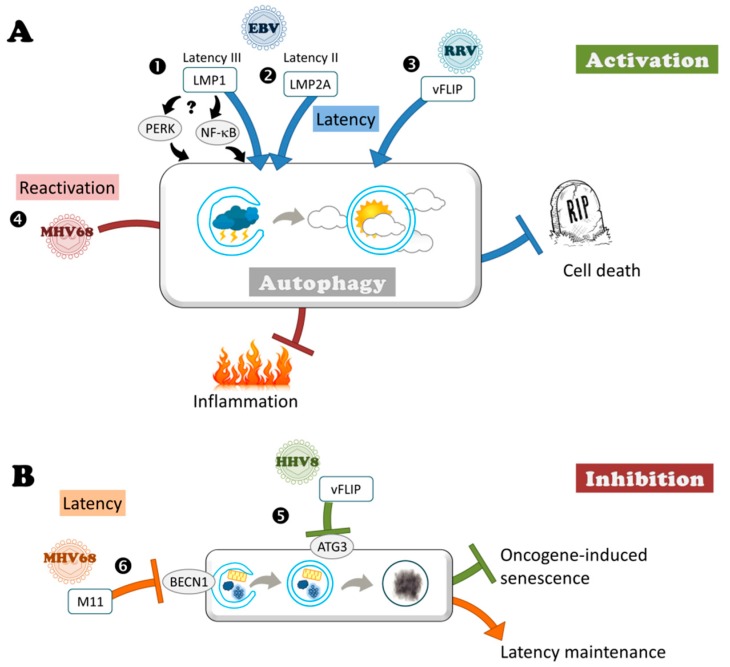
Subversion of autophagy during latency. (**A**) Herpesviruses can benefit from autophagy during latency. ➊ LMP1 encoded by EBV during latency III ➋ LMP2A during latency II, similarly to ➌ vFLIP encoded by RRV protect cell against death by stimulating autophagy (blue T bars), contributing to viral persistence and tumorigenesis. LMP1 induces autophagy either via PERK or via NF-κB. ➍ MHV68 benefits from autophagy to reactivate by limiting virus-induced inflammation (red T bar). (**B**) Herpesviruses can block autophagy during latency. ➎ HHV8 via vFLIP blocks autophagy to protect against oncogene-induced senescence (green T bar). ➏ The inhibition of autophagy by M11 encoded by MHV68 contributes to the maintenance of latent reservoir (orange T bar).

LMP1 is not the only latent protein stimulating autophagy (Figure 3). Indeed, it has been reported that expression of LMP2A in epithelial cells can progressively induce autophagosome formation [96]. EBV can contribute to the development of various epithelial malignancies, including nasopharyngeal carcinoma, associated with latency II program and LMP2A expression. In EBV-positive cells, LMP2A is able, by ERK activation, to block anoikis, a form of programmed cell death induced by inadequate or inappropriate cell-matrix interactions [97]. Fotheringham and collaborators use a model of epithelial cells grown in three dimensional cultures, which form small acini. Expression of LMP2A in this model induces accumulation of autophagosomes and increases expression of autophagy proteins after 10 days of culture [96]. They demonstrate that autophagy impedes formation of the characteristic hollow lumen by promoting resistance to anoikis cell death. Treatment by 3-MA or chloroquine, which inhibits autophagy, increases apoptosis and caspase 3 activation and restores acinus formation.

An anti-apoptotic protein of Rhesus monkey rhadinovirus (RRV) stimulates autophagy during latent infection in order to prevent cell death. Autophagy induction has also a positive effect during the latency of RRV, a virus that naturally infects rhesus monkeys and is closely related to HHV8 [98]. During latency, this virus encodes a homolog of cellular FLIP (FLICE-like inhibitory protein), vFLIP, which activates autophagy (Figure 3). Under pro-apoptotic treatment, ectopic expression of vFLIP enhances autophagosome formation, and also inhibits apoptosis. Probably because autophagy acts as a pro-survival mechanism, vFLIP protects the cells against apoptosis during RRV latent infection but the mechanism of action of vFLIP regarding autophagy is currently unknown. Although initially identified in various viruses, mammals encode several homologs of vFLIP, and these cellular FLIP consist of distinct spliced variants. In contrast to RRV vFLIP, the long and short forms of cFLIP suppress autophagosome formation, acting directly on the autophagy machinery by preventing ATG3 from binding and processing LC3 [52]. Similarly to cFLIP but in contrast to RRV, HHV8 also encodes a vFLIP homolog, which inhibits autophagy by interacting with ATG3. The difference between RRV and HHV8 may be related to the fact that the stimulation of autophagy during latent HHV8 infection has a negative effect on the proliferation of infected cells [99]. Indeed, autophagy triggers oncogene-induced senescence, a robust and sustained anti-proliferative response that restrains cancer progression. This senescence is activated by another viral latency protein known as v-cyclin, which has been found to be capable of activating autophagy. V-cyclin induces the transcription of several autophagy genes (ULK1 (Unc-51 like autophagy activating kinase 1), ATG7, LC3), and leads to an inhibition of mTOR (mammalian Target of Rapamycin), which allows the activation of autophagy. The senescence induced by v-cyclin activation of autophagy is counteracted by vFLIP.

Autophagy can be also modulated during the initiation of HHV8 reactivation. The viral protein RTA (Replication and Transcription Activator) is a transcriptional factor necessary for the lytic reactivation and, moreover, it is also able to activate autophagy [81]. Wen and collaborators observed that autophagy is stimulated during HHV8 reactivation, and that RTA alone induces autophagosome formation in both 293T and B cells. Furthermore, autophagy inhibition affects HHV8 lytic reactivation, suggesting that autophagy probably plays an important role during this step in the virus life cycle. One possible hypothesis for the mechanism of autophagy activation by RTA might be an upregulation of autophagic gene expression, as an increase of BECN1 expression has been observed during HHV8 reactivation.

Autophagy can nip inflammation in the bud to allow MHV68 reactivation. A recent study demonstrated for the first time the beneficial role of autophagy for Herpesvirus in vivo during latency [100]. This was observed in the context of infection of mice with the murine gammaherpesvirus MHV68, which results in lymphoproliferative diseases, similarly to human gammaherpesvirus EBV and HHV8. MHV68 latency occurs in peritoneal macrophages and splenic B cells. Systemic inflammation, and in particular interferon-γ (IFN-γ), impedes reactivation of MHV68 from latently-infected macrophages [101]. Maintaining a low level of inflammation is therefore necessary to allow the reactivation of the virus. Autophagy, by limiting virus-induced inflammation, enhances reactivation from latency. Park and collaborators observed that deficiency in multiple autophagy genes, such as ATG3, ATG5, ATG7, BECN1, or ATG16 in the myeloid compartment decrease the reactivation rate. However, lack of ATG4b gene does not hamper MHV68 reactivation, but this discrepancy may be related to the presence of different isoforms of ATG4 acting on LC3 conjugation. Although MHV68 also establishes latency in splenic B cells, ATG5 deficiency in B cells had no effect on reactivation. Promotion of reactivation by autophagy is due to a decreased production of IFN-γ by T cells (even if this cytokine is not the sole contributor) rather than a direct action on viral replication or on the establishment of latency. Indeed, no evidence for a cell-intrinsic role for ATG genes in MHV68 infection was observed and viral multiplication during early lytic cycle was identical during the first week of infection in peritoneal cells and in macrophages. It would be simplistic to think that by decreasing the autophagy level, the host could boost the inflammation level and, therefore, potentially block any reactivation of these latent viruses. However, this would be at the expense of the organism, with serious complications following a chronic inflammation. It would be simplistic to think that by decreasing autophagy levels, the host could boost inflammation levels and, therefore, potentially block any reactivation of latent viruses. However, this would be at the expense of the organism, with serious complications following chronic inflammation.

Despite this, during latency MHV68 expresses a viral homolog of Bcl-2, named M11, that blocks autophagy by interaction with BECN1 [102,103]. M11 has anti-autophagic and anti-apoptotic activities. Moreover, inhibition of autophagy by M11 plays a role in the maintenance of latency although it has no appreciable impact on the establishment of latency [104]. Indeed, viruses encoding mutant forms of M11 that cannot bind BECN1, but still bind pro-apoptotic Bcl-2 proteins, have a smaller latent virus reservoir, whereas they initially establish latency at levels equivalent to that of WT [104]. The fact that MHV68 encodes an anti-autophagic protein during latency is not necessarily contradictory with a beneficial effect of autophagy on reactivation. Indeed, Park et al. proposed that this beneficial effect is related to non-infected neighboring cells by regulating systemic inflammation, whereas deletion of M11 affects autophagy only in infected cells [100]. Similarly, in a HSV-1 ocular infection murine model, Katzenell and Leib observed induction of autophagosome clusters specifically in bystander non-infected neurons, lacking detectable viral protein expression [105]. The role of these clusters needs to be clarified. Regarding M11, it is possible that the specific inhibition of autophagy by M11 in infected cells is important for maintaining a steady state level of the latency reservoir. The role of M11 anti-autophagic activity may be clarified in the future by infecting autophagy-deficient mice with M11 mutant viruses. If control of autophagy is involved in reservoir maintenance, we could expect to see a decreased reservoir in control animals infected with M11 mutant viruses that are unable to block autophagy, but no difference between WT and mutant viruses in autophagy deficient mice.

## 4. Conclusions

To conclude, evidence accumulated undoubtedly demonstrates that autophagy can be beneficial to several Herpesviruses during lytic cycle and latency as well as reactivation (Table 1). For example, autophagy can protect against cell death and it allows optimal reservoir maintenance during latency [66,98,104]. Production of viral particles can benefit from autophagy which facilitates their transport and their egress [14,15]. Degradation of antiviral components by selective autophagy can also contribute to this phenomenon [91]. However, it is also clear that this “hijacking” depends on the virus, the context of the infection and even the cell types. Moreover, in some specific situations, autophagy remains an antiviral mechanism, by sequestrating viral particles into autophagosomes, by increasing antigen presentation, or by promoting monocyte differentiation, for example [47,54,61]. For several Herpesviruses using autophagy, such as DEV, HCMV, and HSV-2, the precise mechanism of these diversions is still unknown and it will be important in the future to elucidate it, since this can also contribute to improve our knowledge of the autophagic process in general. It is noteworthy that a positive role of autophagy during latency and reactivation has been described only for gammaherpesviruses, whereas no studies have reported similar findings for viruses of the *Alphaherpesvirinae* and *Betaherpesvirinae* subfamilies (Table 1). This raises the question as to whether it is related to virus-induced cell immortalization and oncogenic transformation and needs to be clarified by additional studies. Additionally, no role has been attributed to autophagy for the establishment of latency until now, but its participation can be specific for certain Herpesviruses. Another puzzling finding is the presence of proteins of the autophagic machinery in the extracellular viral particle, suggesting that autophagy could be involved in the secondary envelopment of the tegumented-nucleocapsid into the cytoplasm.

Finally, in the light of this discussion, therapeutic approaches against Herpesvirus-associated pathologies, in particular virus-associated tumors, with the aim of modulating the autophagic process, seem an attractive concept, although probably context-dependent. Treatment of EBV-positive B cells with chloroquine, to block autophagic degradation, potentiates the cell death induced by Nutlin 3, a p53 inhibitor which has been shown to inhibit tumor growth in preclinical studies [66]. As a matter of fact, there have been several studies indicating that autophagy promotes resistance of established tumors to chemotherapeutic agents and that autophagy inhibition by drugs, such as chloroquine or 3-methyladenine, sensitizes tumor cells to cell death induced by cytotoxic therapy [106,107].

Conversely, several studies were performed to examine the possibility of upregulating autophagy in the context of infection. A peptide designed to specifically block the interaction between the MHV68-encoded homolog of Bcl-2 M11 and BECN1 (therefore, preventing M11-mediated downregulation of autophagy) could be used to both restore the tumor suppressor activity of BECN1 and to limit the reservoir of viral latency [103]. Similarly, HHV8 needs to block autophagy to disrupt oncogene-induced senescence and a treatment which leads to autophagy stimulation could increase tumor cell death and therefore could represent an interesting strategy for developing therapies against HHV8-associated tumors [108]. Rapamycin, a classical inducer of autophagy, effectively reduces cell growth of HHV8-infected BCBL-1 lymphoma cell line by promoting autophagy-dependent cell death [52]. Treatment with a FLIP-derived short peptide, which efficiently sequesters FLIP from ATG3 and leads to high levels of autophagy by facilitating ATG3-LC3 interaction, also results in robust cell death with autophagy [52]. Although the direct link with autophagy needs to be confirmed, experimental evidence demonstrates that rapamycin treatment is effective against EBV-associated lymphomas, EBV-associated post-transplant lymphoma disorder (PTLD), and HHV8-associated tumors [108].

## Figures and Tables

**Table 1:** Subversion of Autophagy by Herpesviruses.

Subfamily	Name	Abbreviation	Host	Subversion of Autophagy	Ref.
*Alphaherpesvirinae*	Herpes simplex virus type 1	HSV-1	Human	Transient activation of autophagy in THP-1 cells via MyD88 adaptor protein, beneficial for viral entry	[67]
	Herpes simplex virus type 2	HSV-2	Human	Basal autophagy promotes viral replication in fibroblasts	[70]
	Varicella-Zoster virus	VZV	Human	VZV stimulates complete autophagy in several cell types and that is necessary for efficient viral glycoprotein processing. Hijack of amphisomes for viral egress.	[71,86,87,90] [15]
	Duck enteritis virus	DEV	Waterfowl	Autophagy is stimulated at late time of infection to optimize viral production.	[72]
*Betaherpesvirinae*	Human Cytomegalovirus	HCMV	Human	Infection stimulates autophagy and subsequently blocks autophagosome degradation. Autophagy proteins or membranes participate in viral propagation.	[49,50,74]
	Murine Cytomegalovirus	MCMV	Mouse	The viral protein M45 targets NEMO to autophagosomes for selective degradation and therefore participates to the inhibition of innate immunity	[91]
*Gammaherpesvirinae*	Epstein-Barr virus	EBV	Human	During lytic cycle: autophagic flux is blocked and autophagic vacuoles are hijacked by the virus for envelopment/egress During latency: autophagy stimulation by LMP1 and LMP2A favors cell survival	[14,78] [66,95,96]
	Kaposi’s sarcoma-associated Herpesvirus	KSHV or HHV8	Human	Evidence of viral particle transport in autophagosomes and positive role of autophagy during viral reactivation During latency: Autophagy inhibition blocks oncogene-induced senescence	[80,81] [99]
	Rhesus monkey Rhadinovirus	RRV	Rhesus monkey	During latency: vFLIP-induced autophagy protects cells from apoptosis	[98]
	Murid Herpesvirus 68	MHV68	Mouse and small rodents	Autophagy participates in the control of inflammation, allowing virus reactivation from latency	[100]
